# Immunotherapy of membranous nephropathy: strategic evolution from CD20 monoclonal antibodies to future vaccines

**DOI:** 10.3389/fimmu.2026.1955727

**Published:** 2026-09-15

**Authors:** Kena Yu, Xin Xu, Junwei Gao, Jie Xing, Xiaofan Yin, Qin Song, Kaizhi Wen, Xiaomeng Lin, Xudong Cai, Guanghui Zhong

**Affiliations:** 1 Department of Nephrology, Ningbo Municipal Hospital of Traditional Chinese Medicine (TCM), Affiliated Hospital of Zhejiang Chinese Medical University, Ningbo, China; 2 Department of Nephrology, Affiliated Hospital of Nanjing University of Chinese Medicine, Nanjing, China; 3 Ningbo Research Institute of Chinese Medicine, Ningbo Municipal Hospital of Traditional Chinese Medicine (TCM), Affiliated Hospital of Zhejiang Chinese Medical University, Ningbo, China

**Keywords:** anti-CD20 antibody, B cells, CAAR-T, immune tolerance, immunotherapy, membranous nephropathy, regulatory T cells, vaccine

## Abstract

Membranous nephropathy (MN) arises from autoantibody-mediated attack on podocyte antigens, with immune deposits forming subepithelially at the glomerular basement membrane to trigger complement activation and subsequent podocyte damage, eventually manifesting as proteinuria. Traditional therapy hinges on nonspecific immunosuppression, which is characterized by modest effectiveness and a substantial side-effect profile. In recent years, CD20-targeting monoclonal antibodies, most notably rituximab (RTX), have curtailed autoantibody production through peripheral B-cell depletion and consequently emerged as a first-line treatment option for patients at intermediate-to-high risk. However, a considerable proportion of patients still show no initial response or later relapse. Resistance mechanisms are heterogeneous, encompassing Fc receptor polymorphisms, plasma cell longevity, and anti-RTX antibody formation. To address these hurdles, novel biologics that target the BAFF/APRIL signaling axis, CD38-positive plasma cells, and the complement cascade have entered the clinical arena, further broadening the immunotherapeutic landscape for MN. In parallel, as our understanding of immune memory and autoantibody-generating cells continues to expand, a so-called “immune reset” approach is gaining traction, with its core rationale being the restoration of a robust yet self-tolerant immune system. This review charts the progression of MN therapies, from CD20 monoclonal antibodies and their optimized use, through novel biologics, to vaccine-based strategies that target autoantibody-specific B or T cells (including CAAR-T, Treg therapies, B-cell epitope vaccines, and T-cell vaccines). We further consider their theoretical promise for inducing antigen-specific immune tolerance and the barriers to clinical implementation.

## 1 Introduction

Membranous nephropathy (MN), a leading cause of adult nephrotic syndrome, is driven by circulating autoantibodies that target podocyte antigens and deposit as immune complexes *in situ* along the glomerular basement membrane. This event triggers the complement cascade, which in turn damages podocytes and ultimately manifests as proteinuria (1). In idiopathic MN, anti-PLA2R autoantibodies occur in about 70-80% of patients, correlate with clinical activity, and have become standard for both diagnosis and longitudinal surveillance (1, 2). High-throughput techniques like laser microdissection with mass spectrometry have expanded the MN antigen spectrum, identifying new podocyte targets—THSD7A (3), NELL1 (4), HTRA1, and CNTN1—that underpin precision diagnosis and targeted therapy (5).

For moderate-to-high-risk MN, standard therapy combines glucocorticoids with either cyclophosphamide or CNIs (e.g., cyclosporine A, tacrolimus), but these regimens carry risks of drug dependence, myelosuppression, infection, and nephrotoxicity (6–8). Thus, there exists a pressing clinical gap for immunomodulatory therapies that are both more targeted and less toxic. Given that B cells serve as key drivers of autoantibody formation (9, 10), RTX—a CD20-targeting chimeric monoclonal antibody—eliminates circulating B cells, thereby effecting a significant decline in anti-PLA2R antibody levels and inducing remission. This agent has accordingly become a first-line therapeutic choice, offering better tolerability than conventional therapies (9, 11, 12). Yet these CD20-negative long-lived plasma cells, which are not depleted by RTX, continue to reside in patients and are responsible for both initial treatment failure and subsequent recurrences (9, 13). Other nodes in the MN pathogenic cascade are also druggable. Anti-CD38 antibodies (daratumumab, felzartamab) eliminate RTX-resistant long-lived plasma cells (14, 15); complement blockers against C5, C5aR, or C1s interrupt effector steps at podocytes (9, 16). Belimumab or telitacicept adds another layer by limiting autoreactive B-cell renewal via BAFF/APRIL (17, 18). Yet none is antigen-specific—each broadly dampens immune pathways, carries infection risks, and fails to restore tolerance.

Growing insights into immune memory and the cells that generate autoantibodies are now paving the way for a more forward-looking therapeutic strategy—one that has come to be known as “immune conditioning (19).” The essence of the proposal is to re-establish a stable, self-tolerant immune system—an “immune reset” (20) in short—and this marks a decisive turn in MN therapy: from passive immunosuppression to proactive immune regulation. From this vantage, MN immunotherapy evolves across three escalating tiers. At the base, passive immune clearance—via CD20 antibodies such as rituximab—depletes circulating B cells and curtails autoantibody output, though rebound of the B-cell compartment often reignites disease. One tier up, immune modulation deploys inhibitors of BAFF/APRIL signaling, CD38-positive plasma cells, or the complement cascade not to ablate but to recalibrate immune effector pathways, trading antigen specificity for broader, more sustained control. At the apex, antigen-specific tolerance—the strategic endpoint of MN therapy—is pursued through CAAR-T cells, regulatory T-cell approaches, and B-cell epitope vaccines that restore durable tolerance toward defined autoantigens. Along this logic, the present review traces the full arc of MN immunotherapy: from the clinical deployment and mechanistic refinement of anti-CD20 agents, through biologics targeting plasma cells, survival signals, and complement, to cell- and vaccine-based strategies against autoantibody-specific B or T cells (**Figures 1**, **2**). We critically examine their theoretical rationale, summarize existing preclinical findings, and identify the key challenges that stand in the way of translational application.

**Figure 1:**
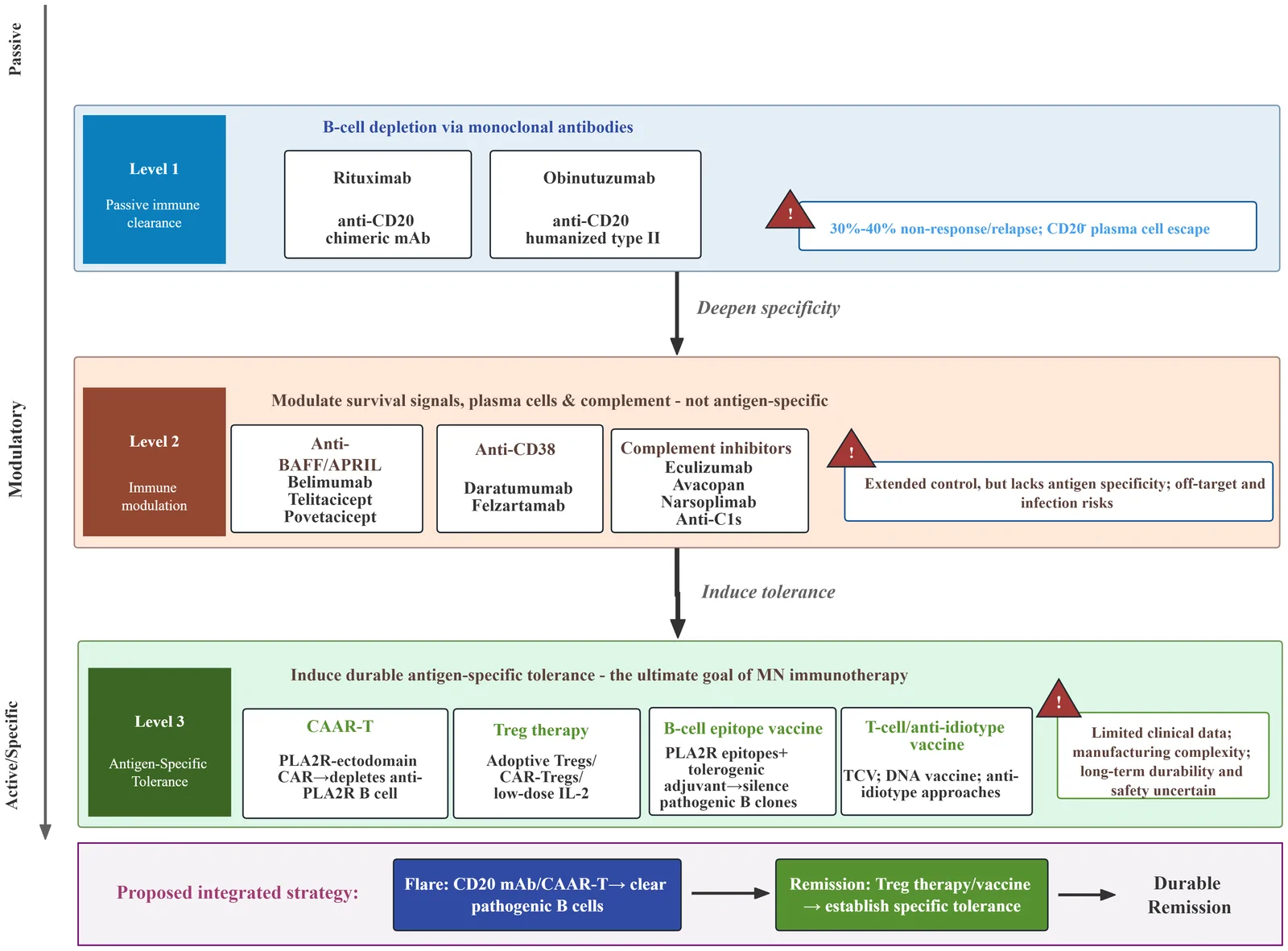
Strategic evolution of membranous nephropathy immunotherapy: from passive immune clearance to antigen-specific tolerance. Level 1: Passive immune clearance. Anti-CD20 mAbs deplete B cells; Level 2: Immune modulation. Non-specific control via BAFF/APRIL blockade, anti-CD38, and complement inhibitors; Level 3: Antigen-specific tolerance. CAAR-T, Treg therapy, epitope vaccines, and T-cell/anti-idiotype vaccines to induce durable tolerance.

**Figure 2:**
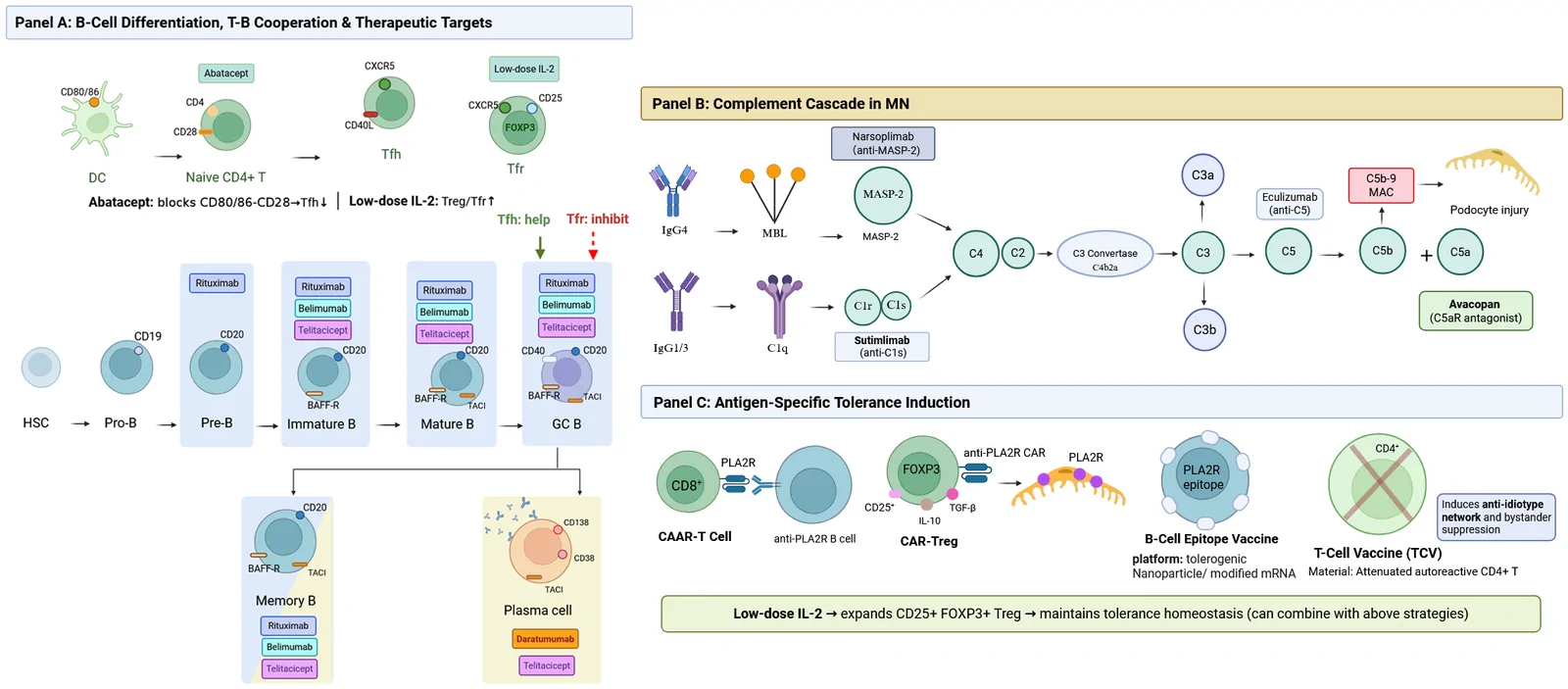
Cellular and molecular mechanisms of immunotherapy in membranous nephropathy. **(A)** DC-primed Tfh activate B cells via CD40L–CD40; Tfr suppress this axis. Therapeutics mapped along B-cell differentiation: abatacept (CD80/86–CD28), low-dose IL-2 (Tregs), rituximab (CD20^+^ B cells), belimumab/telitacicept (BAFF/APRIL), daratumumab (CD38^+^ plasma cells); **(B)** IgG4 triggers the lectin pathway (MBL) and IgG1/3 the classical pathway (C1q), converging on C3/C5 to generate MAC and C5a. Blocked by narsoplimab, sutimlimab, eculizumab, and avacopan (C5aR); **(C)** PLA2R-specific tolerance strategies: CAAR-T (B-cell elimination), CAR-Treg (local suppression), epitope vaccines (nanoparticle/mRNA), T-cell vaccines (anti-idiotype). Low-dose IL-2 expands Tregs to maintain tolerance homeostasis and may combine with the above strategies.

## 2 Immunopathological mechanisms and therapeutic targets in membranous nephropathy

### 2.1 B-cell differentiation, antibody generation, and the limits of anti-CD20 therapy

The immunopathology of MN hinges on the transition of autoreactive B cells into antibody-secreting plasma cells. Along the B-cell developmental trajectory, CD20 is expressed from the pre-B stage through the memory stage, and is absent at the plasma-cell endpoint. This feature defines the practical ceiling for RTX and other anti-CD20 monoclonal antibodies (21, 22). In MN, autoreactive B cells specific for podocyte antigens like PLA2R undergo somatic hypermutation and affinity maturation, then become long-lived plasma cells that settle in bone marrow, spleen, and other secondary lymphoid tissues, where they sustain ongoing production of high-affinity autoantibodies (9, 23). These long-lived plasma cells, however, express no CD20, so they can evade elimination by RTX—a feature that constitutes a principal source of disease recurrence (13). Beyond their role in antibody production, B cells also function as antigen-presenting cells, shuttling podocyte antigens to CD4^+^ T cells and jointly promoting the propagation and maintenance of autoimmune responses (24). This finding places renewed emphasis on the diverse pathogenic roles that B cells play in MN.

### 2.2 The BAFF/APRIL signaling axis: sustaining plasma cell survival and persistence

Multiple cytokines and signaling cascades cooperate to regulate B-cell survival, differentiation, and isotype switching. B-cell activating factor (BAFF) and a proliferation-inducing ligand (APRIL)—key TNF-superfamily ligands—bind to receptors on B cells, thereby promoting cellular viability, facilitating plasma-cell generation, and maintaining their long-term persistence (25). MN patients exhibit significantly higher serum BAFF and APRIL levels, which correlate positively with anti-PLA2R titers and disease activity (18), thereby highlighting the BAFF/APRIL signaling pathway as a key therapeutic target in this setting.

### 2.3 Complement cascade activation and podocyte injury

Complement activation, a central effector event in MN-induced podocyte injury, is initiated by immune complexes formed after autoantibody engagement with podocyte antigens. These complexes activate the classical and lectin cascades, culminating in the generation of C3b, C5b-9, and other active complement fragments (26). Podocyte injury in MN is mediated in part by the membrane attack complex (MAC) C5b-9, which integrates into the cell membrane (26, 27), and is further compounded by C3a- and C5a-driven inflammatory cell recruitment and local immune amplification through receptor engagement (27). Thus, complement inhibition stands out as a principal strategy for MN (28).

## 3.Anti-CD20 monoclonal antibodies as first-line immunotherapy: achievements and constraints

### 3.1 Comparative efficacy and clinical evidence for CD20-targeting agents

Rituximab (RTX), the first chimeric monoclonal antibody targeting CD20, has secured its position in MN therapy through multiple high-quality clinical trials. The GEMRITUX study was the first RCT to demonstrate a significantly higher 6-month remission rate with RTX than with conservative management (11). The MENTOR trial subsequently established RTX as first-line for intermediate-to-high-risk IMN, demonstrating superior 12-month remission over cyclosporine and a widened benefit at 24 months (12). RI-CYCLO showed no significant remission difference between RTX and alternating cyclophosphamide, yet RTX was safer (29). Meta-analysis (30) confirmed a 58% overall remission rate for RTX in MN, with complete remission increasing over longer follow-up.

Obinutuzumab, a humanized type II anti-CD20 mAb, features optimized Fc glycoengineering that enhances antibody-dependent cell-mediated cytotoxicity (ADCC) and direct cell death induction (31). A phase III RCT confirmed obinutuzumab’s superiority over tacrolimus in complete remission, faster response, and durability in primary MN (32), and further showed efficacy in patients resistant to RTX, implying that its strengthened effector functions could circumvent some resistance (33).

### 3.2 Limitations, resistance mechanisms, and strategies to overcome Anti-CD20 resistance

Despite their efficacy in MN, CD20 mAbs face real-world challenges. First, about 30-40% of patients are non-responders or relapse after initial RTX therapy (9, 13). Additionally, residual CD20-negative plasma cells in tissues continue producing autoantibodies after B-cell depletion, resulting in slow anti-PLA2R titer decline or persistent positivity, which correlates with relapse (13).

Anti-rituximab antibodies (ARA) constitute another mechanism of RTX failure. In Allinovi et al. (34), 47% of patients were ARA-positive post-RTX, with a 1-year remission rate of 54% versus 87% in ARA-negative patients, and a significantly higher relapse risk. ARA neutralizes RTX’s antigen-binding site, accelerating drug clearance and weakening B-cell depletion, thereby causing treatment failure (34). Precursors in sanctuary sites (spleen, bone marrow) drive rapid B-cell reconstitution after depletion, and some repopulated cells may adopt a more pathogenic memory phenotype, contributing to relapse (9). To counter these resistance mechanisms, options include switching to obinutuzumab or adding a proteasome or complement inhibitor, which may overcome RTX resistance and improve long-term remission (33, 35).

## 4 In-depth intervention on the B-cell lineage: plasma cell depletion and survival signal modulation

### 4.1 Targeting the BAFF/APRIL signaling pathway

As noted in Section 2.2, BAFF and APRIL are elevated in MN and track with disease severity; pharmacologic blockade of this axis targets the survival signals that keep autoreactive B cells alive. Belimumab, a human IgG1 mAb, binds soluble BAFF, blocks its receptor engagement, and suppresses B-cell survival and differentiation (18). While belimumab alone has modest effects in MN, it synergizes with RTX (SLE-derived evidence) by inhibiting BAFF-mediated B-cell repopulation after RTX depletion, thus prolonging depletion and cutting relapse risk—a strategy based on the BAFF surge post-RTX that would otherwise drive pathogenic B-cell recovery (36). Therefore, the above treatment paradigms in SLE may offer new insights for the management of MN. Unlike single-target belimumab, telitacicept should inhibit a wider range of B-cell activities, notably more potent against APRIL-driven plasma cell viability and antibody production (37). Currently, clinical trials of telitacicept for MN are ongoing, with early results indicating favorable efficacy and safety. Povetacicept (ALPN-303), a next-generation dual BAFF/APRIL inhibitor, was shown in the RUBY-3 trial to reduce proteinuria and preserve renal function in both IgA nephropathy and primary membranous nephropathy. At 48 weeks, proteinuria in primary MN fell by 82% from baseline, supporting this agent as a novel option for targeted immunotherapy in MN (38).

### 4.2 Anti-CD38 monoclonal antibodies: mechanisms and applications in targeting plasma cells

CD38, a type II transmembrane glycoprotein on plasma cells, is preferentially overexpressed on long-lived plasma cells, making it a rational target for their selective ablation (9, 39). Daratumumab is a human IgG1/κ anti-CD38 monoclonal antibody whose application in autoimmune disorders has gradually broadened. It eliminates CD38^+^ plasma cells through ADCC and complement-dependent cytotoxicity (CDC), enhances their phagocytic elimination by macrophages via antibody-dependent cellular phagocytosis (ADCP), and regulates CD38 enzyme activity to affect calcium signaling and NAD^+^ metabolism (40).

In MN therapy, daratumumab confers a theoretical benefit by clearing CD20^-^ long-lived plasma cells that RTX spares, thereby curbing sustained autoantibody output (41). Daratumumab use in RTX-unresponsive MN has been documented only in individual instances, lacking systematic study. However, CD38 expression on multiple lineages (erythrocytes, T/B/NK cells) raises off-target concerns for daratumumab (40). Ongoing MN trials have yet to establish long-term efficacy and safety.

Felzartamab, another human anti-CD38 mAb, eliminates CD38^+^ plasma cells via ADCC and ADCP. Rovin et al. (15) in a phase Ib/IIa study on anti-PLA2R+ MN: 76.9% immunologic responders, 34.6% partial proteinuria remission, no durable hypogammaglobulinemia, and low infection—indicating a safety edge over daratumumab. In contrast, Trillini et al. (42) found good safety in a prospective RTX-resistant cohort, yet without appreciable clinical remission, underscoring the need for larger RCTs to validate efficacy in this subgroup.

### 4.3 Complement-targeted therapy

Complement activation is a key effector of podocyte injury in MN, and inhibitors targeting distinct components of the cascade offer an additional therapeutic dimension (43). Eculizumab, a humanized anti-C5 antibody, blocks C5 cleavage into C5a and C5b, thus preventing membrane attack complex (C5b-9) formation. Eculizumab shows mixed efficacy in MN—some refractory patients derive benefit, but overall responses are low, owing to complement activation variability and sustained upstream classical signaling (44). Avacopan (CCX168) is an oral C5aR antagonist that attenuates complement-induced injury via C5a-directed chemotaxis and leukocyte activation; it has demonstrated steroid-sparing promise in ANCA-associated vasculitis (45), though the phase 3 data supporting this have since been retracted (46); its application in MN is still being investigated. Targeting MASP-2, narsoplimab (OMS721) suppresses lectin-pathway complement and has shown proteinuria reduction in IgA nephropathy, with investigation in MN ongoing (9, 47).

Anti-C1s antibody (e.g., sutimlimab), by blocking C1s to abort immune-complex-driven complement activation upstream, offers a theoretical rationale for MN and similar conditions (16, 48); its optimal use in MN remains to be established (49).

## 5 Antigen-specific interventions: paving the way to immune tolerance

### 5.1 Chimeric autoantibody receptor T-cell therapy

As a CAR-T extension into autoimmunity, CAAR-T therapy links an autoantigen (e.g., PLA2R) ectodomain to T-cell signaling moieties, enabling targeted elimination of B cells bearing cognate autoantibodies. Distinct from tumor-directed CAR-T, CAAR-T relies on autoantigen–antibody pairing to achieve selective removal of pathogenic clones without compromising normal B-cell immunity (50). Antigen recognition requires an ectodomain that retains native conformational epitopes. The full-length PLA2R ectodomain fulfills this requirement; adapting the approach to NELL1, CNTN1, or EXT1/2 depends on whether each ectodomain folds correctly on the T-cell surface (51). Inducible caspase-9 suicide switches allow pharmacologic elimination of CAAR-T cells if on-target, off-tissue toxicity occurs, while prophylactic tocilizumab or anakinra can mitigate CRS/ICANS (50). Costimulatory domain choice governs T-cell persistence and effector function. 4-1BB promotes sustained survival with lower tonic signaling, whereas CD28 drives stronger but short-lived cytotoxicity (52). Current CAAR-T constructs use 4-1BB-CD3ζ (53), though head-to-head comparisons in autoimmunity are lacking. Using PLA2R ectodomain as the antigen-recognition domain, CAAR-T cells selectively eliminate anti-PLA2R B cells *in vitro* and *in vivo*, lowering antibody titers and proteinuria in PLA2R-MN mice, with high specificity and no effect on non-target B cells. A recent study extended the platform to Graves’ disease, showing that CAAR-T bearing the thyrotropin receptor ectodomain specifically eliminated autoreactive B cells, supporting cross-disease adaptability (54). Yet CAAR-T’s clinical advance in MN is constrained by challenges in: tailoring constructs to other antigens (NELL1, CNTN1, etc.) (51), handling patients with unknown targets, mitigating CRS/ICANS, and enabling tunable and reversible T-cell control (50). CAAR-T also carries logistical burdens: autologous production spans 2–3 weeks of leukapheresis, lentiviral transduction, and GMP-grade expansion. CAAR-T for MN is unlikely to cost less than oncology CAR-T therapies, given the smaller patient pool and fewer economies of scale.

### 5.2 Co-stimulatory blockade and cytokine-directed T-cell modulation

In MN, T cells play helper and regulatory roles in immunopathogenesis; co-stimulatory blockade is a key approach to antigen-specific tolerance. Abatacept (CTLA-4-Ig) interrupts T-cell co-stimulation by competing for CD80/CD86, thereby dampening T-cell-dependent B-cell response (55). Abatacept’s efficacy in MN varies, with some studies reporting decreased proteinuria and antibodies, others not, likely attributable to heterogeneous T-cell-dependent/independent autoantibody mechanisms (56, 57). Additionally, abatacept may act directly on podocytes: by binding B7-1 (CD80) on injured podocytes, it stabilizes β1-integrin and preserves slit diaphragm integrity, as shown in B7-1-positive FSGS (58). Upstream immune inhibition also poses infection and cancer surveillance risks. Given that MN also engages innate immunity and complement (16, 56), sole co-stimulatory targeting may be insufficient.

T-cell differentiation is another target under investigation. In MN, Th17/Treg dysregulation plays a pivotal role—IL-6/IL-23-axis promotes Th17 differentiation and inflammation, whereas Treg deficiency undermines tolerance (59). IL-6 contributes to Th17-mediated inflammation in MN, and IL-6 receptor blockade with tocilizumab—though primarily validated in iMCD-associated nephropathy (60)—represents a mechanistically rational strategy for Th17-high primary MN that awaits clinical validation (61). The complexity of MN pathogenesis calls for caution in T-cell targeting—mono-targeting may not suffice for sustained tolerance, and combinatorial approaches that coordinate innate and adaptive immunity will probably be required (16, 56, 62).

### 5.3 Regulatory T-cell therapy

Tregs are key to peripheral tolerance, restraining effector T-cell and autoreactive B-cell activation/expansion via inhibitory cytokines (IL-10, TGF-β) and cell-contact mechanisms (63). Reduced Treg counts and dysfunction in MN, linked to activity and antibody levels (64), argue for restoring tolerance via adoptive autologous Tregs (ex vivo-expanded) or endogenous expansion to limit disease progression.

CAR-Tregs, engineered to display antigen-specific CARs, deliver precise immunosuppression to target tissues or cells. They can localize to autoantigen-presenting cells or inflamed areas in autoimmunity, providing regional control with fewer systemic adverse effects (65). HLA matching or CRISPR editing improves Treg stability and persistence, reducing MHC-related restrictions and rejection (66). Low-dose IL-2 expands Tregs to rebalance tolerance, with preliminary efficacy in SLE and other autoimmune diseases—suggesting a promising outlook for MN (67–69). Yet key obstacles to Treg therapy include the technical difficulty of expanding and sustaining Tregs ex vivo, their instability in inflamed settings (risking effector T-cell conversion), and limited homing/persistence after adoptive transfer (64).

## 6 Outlook: from passive elimination to active immunization

### 6.1 B-cell epitope-based immunotherapy vaccine

Designed to induce antigen-specific tolerance, B-cell epitope vaccines use epitopes from autoantigens (e.g., PLA2R) with suitable delivery and adjuvants to promote tolerance over activation, thereby achieving durable elimination or silencing of pathogenic B-cell clones. Epitope vaccines offer theoretical benefits over traditional B-cell depletion, including greater specificity, reduced off-target effects, and durable tolerance induction (70, 71). In the HN rat model (72), immune interventions targeting the anti-gp330 (rat Megalin) autoimmune response—such as depleting antibody-producing cells or suppressing autoantibody production—prevented the development of HN and reduced proteinuria (73), supporting the rationale for MN epitope vaccine development. Fine-mapping of human PLA2R B-cell epitopes (74) further enables patient-specific vaccine design based on individual epitope profiles. B-cell epitope vaccines face clinical barriers: epitope diversity in MN antigens may limit a one-size-fits-all approach, demanding patient-tailored design by epitope profile (51, 75); moreover, self-antigen vaccines carry the risk of tolerance breakdown and enhanced autoimmunity, requiring refined dosing and immune surveillance (76, 77). Choosing adjuvants and delivery systems that promote tolerance rather than immunogenicity remains a central hurdle in epitope vaccine design; future work may leverage nanoparticle, DNA, or mRNA platforms with refined antigen presentation and co-stimulation to safely achieve antigen-specific tolerance (78).

### 6.2 Vaccines: T-cell-based and anti-idiotype approaches

T cell vaccination (TCV) uses attenuated or inactivated autologous T cells to elicit immune responses against pathogenic clones, thereby eliminating autoreactive T cells and inducing immune tolerance. Initially validated by Cohen et al. (79) across multiple autoimmune models, this strategy also curbs proteinuria and attenuates glomerular injury in the HN model (80). Wang et al. (81) found that TCV induces CD8^+^ regulatory T cells, which suppress pathogenic T and B cell responses via contact-dependent mechanisms and protect rats from HN challenge. By delivering antigens via CD40 to dendritic cells and evoking tolerogenic responses locally, DNA vaccines have been shown to avert HN (73), providing a proof-of-concept for TCV in MN—yet transitioning to clinical practice hinges on clonotype identification, standardized production, and surveillance of immune status.

Anti-idiotype vaccine strategies are grounded in the characterization of pathogenic autoantibody idiotypes, as demonstrated for anti-gp330 in HN (82). Also, DNA vaccination with a pathogenic TCR-encoding construct triggers specific anti-TCR humoral responses, attenuating proteinuria in active HN (83). Clinical translation of these strategies must account for inter-individual variability in autoantibody and TCR repertoires, likely requiring high-throughput sequencing for personalized vaccine design. Single-cell sequencing and bioinformatics now enable precise identification of pathogenic B-cell clones and TCR sequences, laying the groundwork for individualized anti-idiotype and TCV vaccines (84).

### 6.3 Novel vaccine platforms and technological innovations

Recent vaccine technology advances offer a major opportunity for MN vaccine translation. mRNA-based platforms, characterized by rapid design, scalability, and multi-antigen coding, have proven effective in preventing infectious diseases and are promising for autoimmune tolerance (78, 85, 86). Optimized design and delivery allow efficient autoantigen expression and tolerogenic presentation in APCs, addressing the key drawback of conventional protein vaccines—excessive immunogenicity.

Nanoparticle delivery systems provide a tunable platform for precise antigen delivery and immune modulation. Modulating size, surface chemistry, and composition enables targeted delivery to specific immune subsets, while co-delivering immunomodulators like IL-10 and TGF-β steers responses toward tolerance (87, 88). Moreover, delivering nanoparticles to tolerogenic organs such as the liver and intestinal mucosa could harness their natural tolerogenic milieu to promote antigen-specific Treg induction and expansion.

### 6.4 Challenges and translational prospects

Despite the theoretical promise of MN-based vaccines, clinical translation remains challenging. Target heterogeneity is a major obstacle. Although the antigen repertoire in MN is relatively well established (5), antibody targets vary considerably across patients, and some individuals carry multiple autoantibodies simultaneously, rendering a single-antigen vaccine inadequate for covering the entire patient spectrum. Furthermore, epitope spreading may occur over the disease course, where an initial response against one epitope gradually extends to other epitopes on the same antigen or to distinct antigens, further complicating vaccine design (89).

Moreover, how long tolerance lasts determines the overall efficacy of vaccine strategies. Long-term maintenance after discontinuation is governed by Treg stability, memory response regulation, and immune microenvironment remodeling (64, 80). While CD8^+^ Tregs are key mediators of peptide vaccine–driven tolerance (81, 90), their long-term stability and functional maintenance are poorly understood. On the safety front, self-antigen vaccines pose inherent risks of tolerance disruption and exacerbated autoimmune responses, underscoring the need for rigorous surveillance and control mechanisms (71, 76, 77).

The translational outlook for MN vaccine strategies could be advanced through several avenues: precisely identifying pathogenic B-cell clones and TCR sequences via high-throughput sequencing and bioinformatics to enable personalized vaccines (84, 91); exploring novel platforms such as nanoparticle delivery systems and mRNA vaccines to refine antigen presentation and guide immune responses (92); combining multiple immune interventions—for instance, CAAR-T with Treg therapy, or epitope vaccines with low-dose IL-2—to synergistically foster antigen-specific tolerance (50, 54, 93); and establishing comprehensive biomarker panels for early efficacy prediction and real-time monitoring (94). With rapid advances in immunology and biotechnology, vaccine strategies for MN are poised to move from proof-of-concept to clinical application, offering a new route toward long-term remission and potentially even cure.

Several rational therapies have underperformed in MN: eculizumab, limited by persistent upstream activation; felzartamab, immunologically active yet clinically ineffective in RTX-resistant disease; abatacept, with inconsistent results; and avacopan, whose phase 3 data were retracted. These results underscore that mechanistic rationale alone does not guarantee clinical benefit, and that biomarker-guided patient selection will be essential for future antigen-specific therapies. Personalized vaccines meet comparable obstacles: individualized epitope mapping and per-patient manufacturing restrict widespread use. Pre-formulated multi-epitope mRNA vaccines against prevalent PLA2R epitopes may cut costs, albeit with reduced personalization. Demonstrating cost-effectiveness will be essential for reimbursement.

## 7 Discussion

Here we provide a systematic overview of MN immunotherapy’s development, revealing a clear transition from nonspecific immunosuppression toward antigen-specific immune tolerance that unfolds across three escalating levels.

Vaccinology offers a useful vantage on this tiered progression. The three tiers of MN immunotherapy and the established vaccine paradigm pursue opposite ends—tolerance induction versus protective immunity—yet converge on shared technological platforms. mRNA, nanoparticle carriers, and adjuvant systems, all originally refined for prophylactic vaccines, can be repurposed to build tolerogenic vaccines against MN. The correspondence runs deeper still: passive B-cell depletion mirrors passive antibody transfer; immune modulation recalls the action of immunomodulatory vaccines; and antigen-specific tolerance echoes the defining principle of prophylactic vaccination—antigen-specific protection—while inverting its purpose from immunity to tolerance.

Nevertheless, MN vaccine strategies are still at an early translational stage, and the gap with infectious disease vaccines is substantial. The reasons: the tolerogenic machinery for self-antigens is intrinsically more intricate than anti-foreign immunity, involving both central and peripheral checkpoints; the MN population is small, limiting market pull and making patient enrollment challenging; and safety margins are extremely narrow, as any sign of flare-up could halt further development.

Priorities for future studies include, first, refining self-antigen delivery via nanoparticles or peptide–MHC complexes to enhance tolerogenic vaccine precision, and testing multi-epitope regimens to address MN subtype diversity. Another priority is to develop standardized clinical endpoints and biomarker systems for evaluating MN tolerogenic vaccines. A clearance-to-tolerance sequential regimen merits testing. For clearance, rituximab is first-line; if refractory, obinutuzumab, felzartamab, or CAAR-T (most antigen-specific) can be substituted, with choice guided by anti-PLA2R titer, prior therapy, and ARA status. We propose that the transition to tolerance induction could be guided by: anti-PLA2R titers declining approximately ≥ 50% or reaching seronegativity (13, 94); CD19^+^ B-cell recovery (~6–9 months post-rituximab, extrapolated from SLE experience) (36); and partial remission per KDIGO criteria (proteinuria <3.5 g/24h) (95). These proposed thresholds require prospective validation. Such cutoffs remain provisional pending prospective confirmation. Tolerance induction would ideally rely on PLA2R epitope vaccines carried by tolerogenic nanoparticles or mRNA constructs, pairing them with tolerogenic rather than inflammatory adjuvants. Low-dose IL-2 could additionally support Treg expansion.

## 8 Conclusion

The treatment of MN has shifted from nonspecific immunosuppression toward B-cell depletion and, more recently, antigen-specific modulation. Rituximab, a CD20 antibody, clears circulating B cells and remains first-line, but resistance involving Fc receptor variants, long-lived plasma cells, and ARA production has prompted efforts to target deeper disease mechanisms. New biologics directed against BAFF/APRIL signaling, CD38^+^ plasma cells, or complement provide alternatives for RTX non-responders, though each carries limitations. Strategies such as CAAR-T, Treg therapy, and B-cell epitope vaccines may enable durable remission and represent a shift toward antigen-specific therapy. Their clinical adoption, however, faces challenges including target heterogeneity, durability of tolerance, and safety concerns.

A sequential “clearance-then-tolerance” approach could combine B-cell depletion (CD20 mAbs or CAAR-T) during active disease with Treg therapy or tolerogenic vaccination in remission to sustain antigen-specific tolerance. mRNA and nanoparticle platforms may facilitate clinical translation of these vaccine strategies toward more personalized MN care.
